# CsPbI_3_ Perovskite Quantum Dot-Based WORM Memory Device with Intrinsic Ternary States

**DOI:** 10.1021/acsami.4c07044

**Published:** 2024-07-22

**Authors:** Luhang Xu, Yuang Fu, Yuhao Li, Guodong Zhou, Xinhui Lu

**Affiliations:** † Department of Physics, The Chinese University of Hong Kong, New Territories, Shatin, Hong Kong SAR 999077, China; ‡ Spallation Neutron Source Science Center, Dongguan 523803, China; § College of Integrated Circuits, Zhejiang University, Hangzhou 311200, China

**Keywords:** perovskite quantum dots, write-once-read-many-times memory, intrinsic ternary states, mobile iodine vacancies, conductive filaments, in situ conductive atomic force microscopy

## Abstract

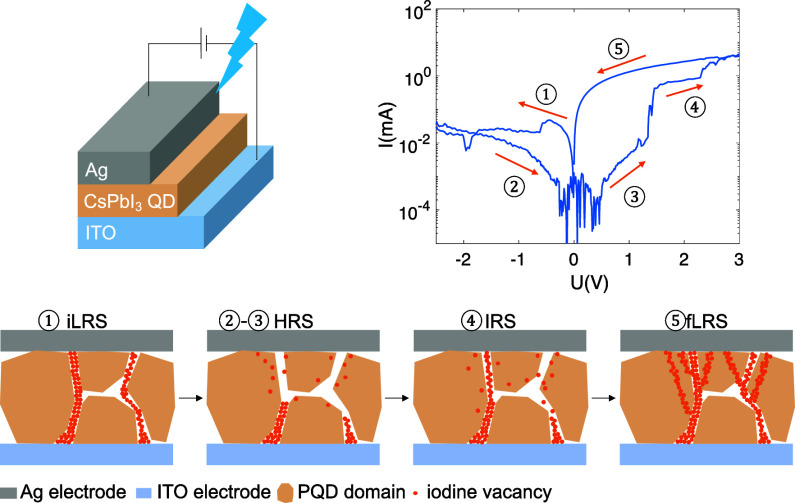

The migration of mobile ionic halide vacancies is usually considered detrimental to the performance and stability of perovskite optoelectronic devices. Taking advantage of this intrinsic feature, we fabricated a CsPbI_3_ perovskite quantum dot (PQD)-based write-once-read-many-times (WORM) memory device with a simple sandwich structure that demonstrates intrinsic ternary states with a high ON/OFF ratio of 10^3^:10^2^:1 and a long retention time of 10^4^ s. Through electrochemical impedance spectroscopy, we proved that the resistive switching is achieved by the migration of mobile iodine vacancies (V_I_s) under an electric field to form conductive filaments (CFs). Using in situ conductive atomic force microscopy, we further revealed that the multilevel property arises from the different activation energies for V_I_s to migrate at grain boundaries and grain interiors, resulting in two distinct pathways for CFs to grow. Our work highlights the potential of CsPbI_3_ PQD-based WORM devices, showcasing intrinsic multilevel properties achieved in a simple device structure by rationally controlling the drift of ionic defects.

## 1 Introduction

The emergence of the Internet of Things (IoTs) necessitates the development of novel memory devices for high-efficiency data storage. Nonvolatile resistive switching (RS) memory devices with a metal–semiconductor–metal sandwich-like structure have attracted extensive attention due to their fast-switching speed, long retention time, and low cost per bit.^1^ By applying a proper electric signal, nonvolatile RS devices can switch between a high resistance state (HRS) and a low resistance state (LRS) by changing the bulk conductivity of the semiconductor active layer and retaining the state after signal removal. In particular, RS devices that permanently retain the LRS after transition, known as the write-once-read-many-times (WORM) memory devices,^2,3^ can protect data from being overwritten or deleted unintentionally, greatly improving data security. Therefore, WORM memory devices are widely employed in the archival storage of images and videos, radio frequency identification (RFID) tags, and noneditable databases.^4^ Multilevel operation is another desirable feature for RS devices as it greatly improves the data processing and storage capabilities of a single RS device.^5^ To regulate the filament formation and maintain several states within the active layer, an extra compliance current (CC) unit is usually added,^5,6^ which inevitably complicates the circuit structure design.^7,8^

Metal halide perovskites (MHPs) are promising active layer materials for perovskite photovoltaics, photodetectors, and light-emitting diodes.^9^ One intrinsic characteristic that limits the performance and stability of those optoelectronic devices is the presence of mobile ionic halide vacancies with low formation (0.1–0.2 eV) and migration (0.1–0.6 eV) energy,^10^ which can drift under an electric field and optical excitation.^9,11−14^ Compared with bulk perovskites, the concentration of mobile ionic vacancies is even higher in perovskite quantum dots (PQDs). This arises from the weak interaction between commonly employed surface ligands and halide ions in perovskite PQDs, resulting in a large amount of surface ionic vacancies as ligands detach during the purification, aging, dilution, and thermal annealing of PQDs.^15−17^

Although the presence of mobile ionic vacancies in perovskite materials is undesirable for many optoelectronic devices, the potential of rationally controlling the migration of ionic vacancies to form conductive filaments (CFs) makes perovskite materials promising for RS devices. Several recent works have demonstrated RS devices with binary resistance states using PQDs, including CsPbBr_3_,^5,18,19^ CsPbCl_3_,^20^ MAPbBr_3_,^21^ FAPbI_3_,^22^ and Rb_6_Pb_5_Cl_16_.^6^ However, the intrinsic multilevel operation has not yet been achieved in PQD-based RS devices. Additionally, the correlation between the PQD active layer morphology and CF formation remains unclear.

In this work, we synthesized CsPbI_3_ PQDs using the hot-injection method^23^ and utilized them for the first time as the active layer in RS devices. The device with a simple Ag/CsPbI_3_ PQDs/indium tin oxide (ITO) sandwich-like structure demonstrated excellent WORM performance and intrinsic multilevel behavior. Without any extra CC unit^5,6^ or optical stimulation,^18,19^ transitions between ternary states with a high ON/OFF ratio of 10^3^:10^2^:1 can be realized solely by electrical bias, and the transited state can be retained for over 10^4^ s after bias removal. Electrochemical impedance spectroscopy (EIS) and temperature-dependent *I*–*V* measurements demonstrated that the RS mechanism originates from the annihilation and formation of CFs made of mobile iodine vacancies (V_I_s). In situ conductive atomic force microscopy (c-AFM) was then used to directly visualize the formation and growth of CFs from PQD grain boundaries (GBs) to grain interiors (GIs), explaining the multilevel property observed in our RS devices. Overall, our work underscores the potential of CsPbI_3_ PQDs for efficient multilevel WORM devices with a simple device structure via the rational control of V_I_ migration.

## 2 Results and Discussion

All-inorganic CsPbI_3_ PQDs were synthesized through a conventional hot-injection method (further details are shown in the Experimental Section).^23^ We incorporated oleic acid (OA) and oleylamine (OAm) as surface ligands to regulate the growth and stabilize the structure of PQDs.^16^ Besides, their weak interaction with iodine-based PQDs allows partial detachment of ligands during film processing.^16,17^ This generates a large population of mobile V_I_s, which can be aligned under an electric field to form CFs, as further discussed below. Those insulating long-chain ligands can also reduce the conductivity of PQD films^24^ in the HRS, which improves the On/Off ratio of the device.

**Figure 1:**
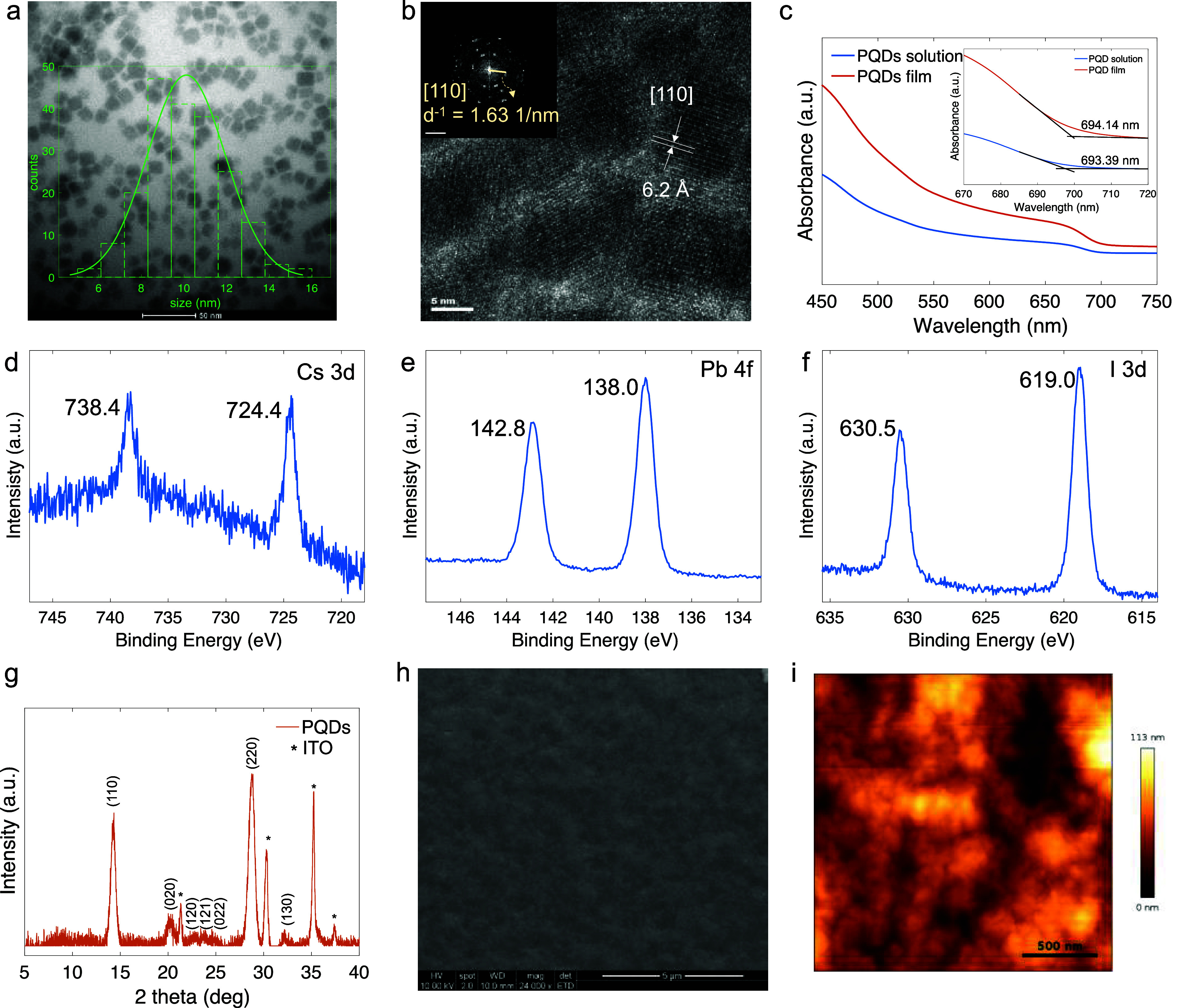
(a) TEM image of CsPbI_3_ PQDs; the inset: the size distribution. (b) the corresponding HRTEM image; the inset: the reciprocal lattice structure obtained by fast Fourier transform (FFT). (c) UV–vis absorption spectra of CsPbI_3_ PQD dispersed in a mixed solvent of hexane and toluene (1:1 vol %) and the thin film; the inset enlarges the wavelength range near the absorption edge and gives the fitting results of the bandgap. XPS spectra of (d) Cs 3d, (e) Pb 4f, and (f) I 3d for annealed CsPbI_3_ PQD film. (g) XRD pattern of annealed CsPbI_3_ PQD film deposited on ITO substrate with the diffraction peaks from perovskite γ-phase and from ITO being labeled. (h) SEM and (i) AFM topography images of the annealed CsPbI_3_ PQD film surface.

Upon synthesis, the PQDs exhibited a cubic shape with an average size of 10 nm, as demonstrated by the transmission electron microscopy (TEM) image (Figure 1a). Clear lattice fringes with an interplanar distance of 6.2 Å were observed in both the high-resolution TEM (HRTEM) image and its fast Fourier transform (FFT) pattern, as depicted in Figure 1b. The bandgap of the CsPbI_3_ PQDs was determined to be around 1.79 eV in both solution and thin film, as derived from ultraviolet–visible (UV–vis) absorption spectra (Figure 1c), consistent with previous findings.^25^ The X-ray photoelectron spectroscopy (XPS) results (Figure 1d–f) present the spin–orbital splitting peaks of Cs, Pb, and I at 724.4 and 738.4 eV, 138.0 and 142.8 eV, 619.0 and 630.5 eV, respectively, consistent with the previously reported values for CsPbI_3_ PQDs.^26^ Additionally, the X-ray diffraction (XRD) pattern of CsPbI_3_ PQDs (Figure 1g) displayed well-defined Bragg peaks originating from the γ-phase of perovskite.^27^ PQDs dispersed in a mixed solvent of toluene and hexane (1:1 vol %) can be deposited to form densely packed thin films with a grain size of around 500 nm, as evidenced by top-view scanning electron microscopy (SEM, Figure 1h) and tapping-mode AFM (Figure 1i) images. The impact of the active layer morphology on the RS device performance will be further discussed below.

**Figure 2:**
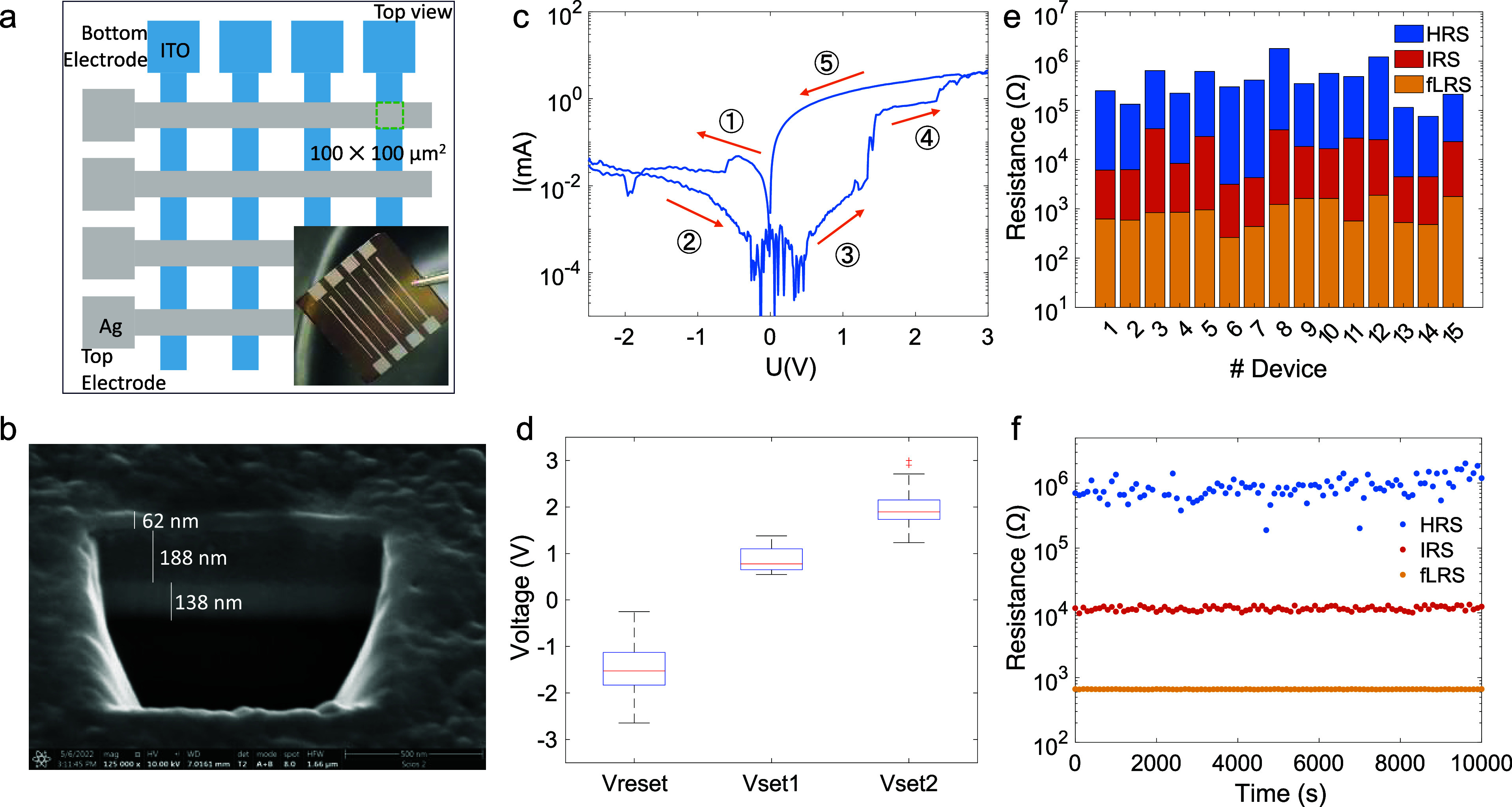
(a) Schematic configuration of the device. The inset: a photograph of the as-prepared device. (b) Cross-sectional SEM image of the entire device stack obtained by FIB. (c) *I*–*V* characteristics of the RS device. The bias (applied to the Ag electrode) was swept from 0 → −2.5 V → 0 → +3 V → 0 without compliance current. (d) Statistical data of voltage distributions for RESET, SET1, and SET2 processes, respectively. (e) Resistances of HRS, IRS, and LRS for 15 independent RS devices. (f) Retention performance of RS devices read by a small reading voltage (0.1 V).

The RS device adopts a simple structure of Ag/CsPbI_3_ PQDs (annealed)/ITO with an effective device area of 100 × 100 μm^2^, defined by the overlap of two electrodes, as illustrated in Figure 2a. The cross-sectional SEM image of the device stack obtained using the focused ion beam (FIB) technique, shown in Figure 2b, clearly reveals a sandwich structure. The thickness of the PQD layer is around 190 nm, deposited from a PQD solution (toluene:hexane 1:1 vol %) with a concentration of 80 mg/mL to optimize device performance (see Figure S1).

To investigate the electric performance of the device, the ITO electrode was grounded, and bias was applied to the Ag electrode. Following the 0–(−2.5)–0–(+3)–0 V sweeping voltage loop, a typical *I*–*V* curve is shown in Figure 2c, showcasing successive RESET and SET processes. Starting from the initial LRS (① iLRS), the device transits to an HRS (②) at around −1 V and maintains this state as voltage scans back to 0 V. Under positive bias, two set processes occur: the first one sets the device to an intermediate resistance state (IRS, ③–④) at 1 V, while the second one sets it to the final low resistance state (fLRS, ④–⑤) at 2 V. Upon scanning back from 3 to −3 V, the device remains in the fLRS instead of reverting to HRS (see Figure S2a). The fLRS is retained after over 100 cycles of *I*–*V* scan (Figure S2b), demonstrating typical characteristics of a WORM-type memory device. The noise level of about 1 μA in the *I*–*V* curve is limited by the source meter (Figure S3). The substantial difference in the set voltages under forward bias (∼1.0 V for V_set1_ and ∼2.0 V for V_set2_, see Figure 2d) ensures the reliability of these two set processes. Device performance statistics are presented in Figure 2e, indicating a current ratio of 10^3^:10^2^:1 for fLRS:IRS:HRS, facilitating specific state differentiation. Additionally, resistances of around 10^6^, 10^4^, 10^3^, and 10^3^ Ω were maintained for over 10^4^ s without noticeable degradation for HRS, IRS, fLRS, and iLRS, respectively, as shown in Figures 2f and S4. Additionally, we note that the Joule heat generated during state transitions of RS devices does not induce the growth or structural change of PQDs, as confirmed by the invariant photoluminescence (PL) peak of PQDs at 690 nm in iLRS and fLRS, as shown in Figure S5.

To measure device stability, we stored RS devices in an N_2_-filled glovebox and observed their performance within the first 10 days after fabrication, as depicted in Figure S6. The *I*–*V* curves (Figure S6a,b) demonstrate well-maintained binary states under a negative bias and ternary states under a positive bias. Figure S6c illustrates the resistances of the three states (HRS, IRS, and fLRS) measured on each day, with the resistance ratio for HRS/fLRS maintained at over 10^2^ even after 10 days. Additionally, the fLRS also remained stable for 10 days following the set process as shown in Figure S6d. Therefore, RS devices based on CsPbI_3_ PQDs prove suitable for WORM-type memory devices, with the iLRS and ternary states controlled solely by electric bias. Unlike previously reported PQD-based RS devices, multilevel operation in our device was achieved without an extra CC unit,^5,6^ which largely simplifies the circuit design for future applications.

**Figure 3:**
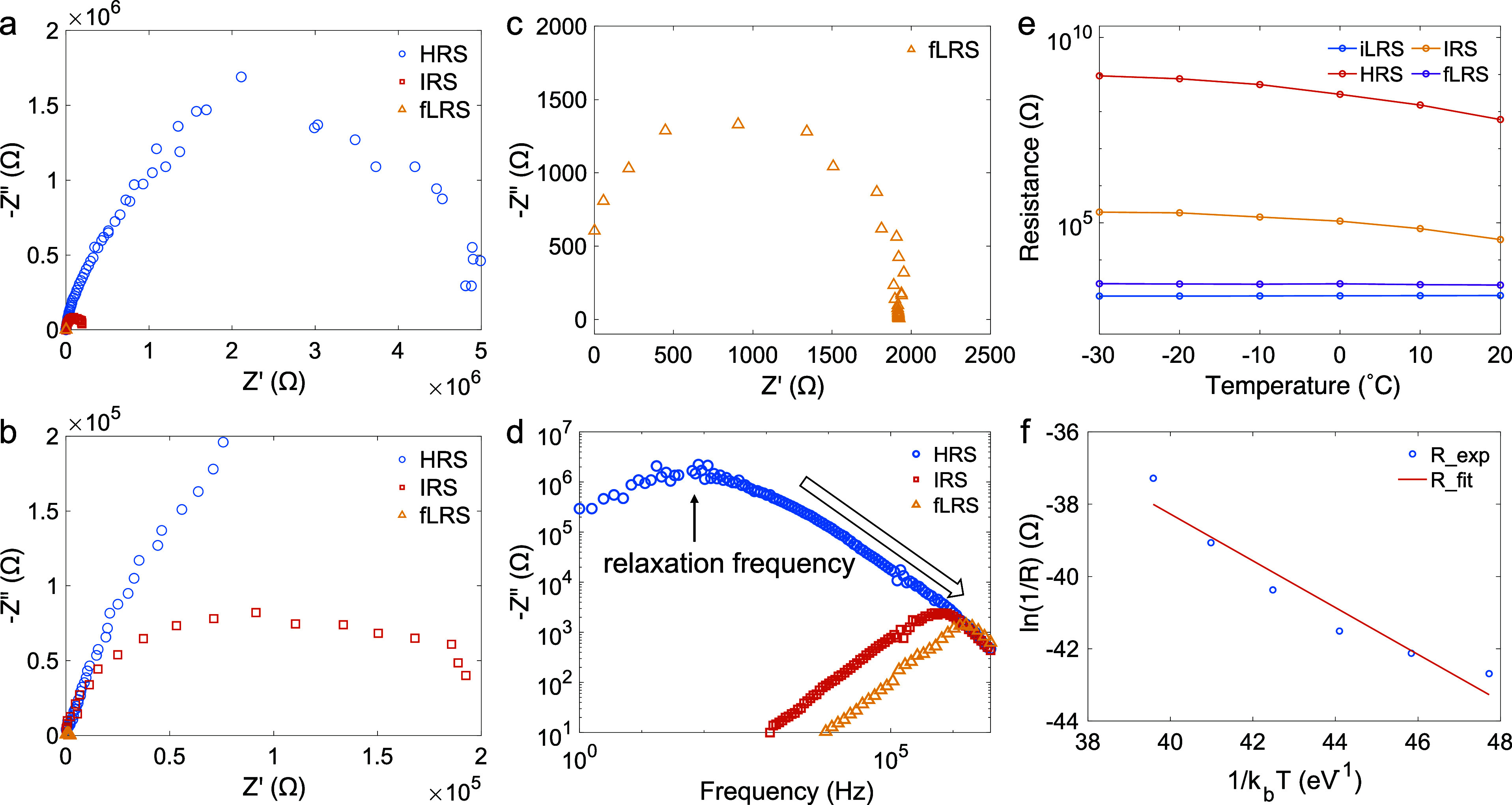
Impedance spectra of the Ag/CsPbI_3_ PQDs/ITO device: (a) Nyquist plots of the HRS, IRS, and fLRS along with the zoomed-in spectra of (b) IRS and (c) fLRS. (d) Bode plot of the imaginary part of the complex impedance for HRS, IRS, and fLRS, respectively, with the change in the relaxation frequency highlighted. (e) Temperature-dependent resistance of iLRS, HRS, IRS, and fLRS, respectively. (f) Arrhenius plot to determine the activation energy for ion migration in the HRS from the fitting.

We then utilized EIS to investigate the RS mechanism of the PQD devices. In general, RS mechanisms can be categorized into interface-type^28^ and filamentary-type^29^ conduction. Under all states, the Nyquist plots of our device (Figure 3a–c) reveal only one major semicircle corresponding to the bulk-limited conduction within the PQD active layer, and its radius decreases monotonically from HRS to LRS. Consistently, there is only one detectable relaxation frequency in the Bode plot (Figure 3d), which gradually shifts to higher values throughout the set process. Those observations rule out the possibility of interface-type conduction, in which case an additional arc would appear in the low-frequency region originating from the perovskite/contact interface and disappear due to the formation of the uniform AgI layer at this interface during the transition from HRS to LRS.^28,30^ Furthermore, we can confirm that the CFs are induced by a valence change mechanism (VCM) instead of electrochemical metallization (ECM). In the latter case, the CFs formed by the diffusion of metal atoms from the electrode into the active layer can be modeled as a resistor connected in series with an inductor after transition to LRS, resulting in a vertical line in the Nyquist plot,^29^ which is not observed for our devices. Additionally, RS devices fabricated using an inert Au top electrode exhibited similar WORM and multilevel behaviors (Figure S7), further ruling out the possibility of ECM-type CFs. We then conducted temperature-dependent resistance measurements under each state to understand the origin of the VCM-type CFs formed in our devices. Figure 3e illustrates the gradual change of the temperature coefficient of resistance (TCR) from the HRS to the LRS induced by CF formation and growth. The negative TCR observed in HRS suggests a semiconductor-type conduction mechanism, with the activation energy determined from the Arrhenius plot (Figure 3f) being 0.32 eV. This aligns with the typical migration energy of V_I_s.^10,31,32^ Throughout the set processes from HRS to IRS and fLRS, the magnitude of TCR decreases and approaches zero, indicating that more V_I_s are formed and accumulate to form CFs with various compositions.^33^ This compositional variation was confirmed using scanning transmission electron microscopy-energy dispersive X-ray spectroscopy (STEM-EDXS). From the cross-section image of the RS device preset to fLRS, two distinct regions can be identified based on the compositional inhomogeneity (Figure S8a–c). The EDXS vertical line scans taken at Region 1 (Figure S8d,e) show a gradual change in the I/Pb ratio, while the Cs/Pb ratio stays constant. By contrast, both the I/Pb and Cs/Pb ratios remain constant in Region 2 (Figure S8f). The local change in the I/Pb ratio signifies the redistribution of V_I_s in Region 1 to form CFs, consistent with the results of EIS and temperature-dependent *I*–*V* measurements.

**Figure 4:**
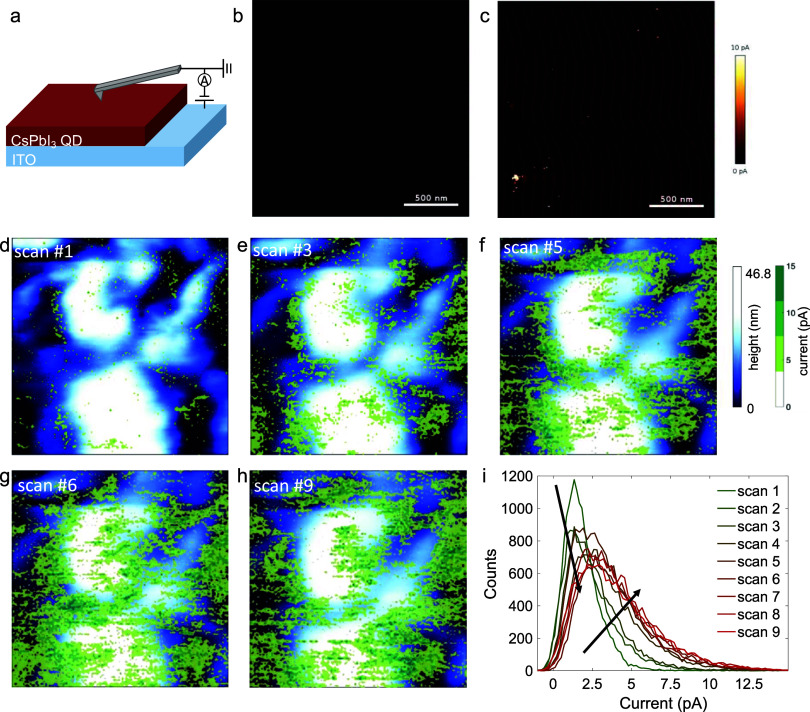
(a) Setup for c-AFM measurements. Ex situ c-AFM images under the −2.5 V reading bias of as-cast (b) and annealed (c) PQD films. The bright spots in (c) are the local high current region due to the presence of CFs. The AFM topography mappings (blue) overlapped with current mappings (green) during the (d) first, (e) third, (f) fifth, (g) sixth, and (h) ninth scans of the in situ c-AFM measurement. (i) Current histogram of each scan.

Having identified the major RS mechanism, we investigated why our devices start from an unusual iLRS instead of the commonly reported initial HRS (iHRS).^1,6−10^ First, we found that the device incorporating an as-cast PQD film exhibits an iHRS under negative bias, as shown in Figure S9, suggesting that thermal annealing of the PQD film is responsible for its unusual iLRS. Although the surface topography of the active layer remains unchanged after thermal annealing (Figures 1i and S10 for annealed and as-cast films, respectively), distinct CF formation processes are revealed by ex situ c-AFM measurements. The experimental setup is shown in Figure 4a, where a grounded Pt-tip was used as the top electrode, and the voltage was applied to the ITO substrate. Taking into account the different geometries of the c-AFM tip and the flat electrodes in actual devices, a thinner film (deposited from 40 mg/mL PQD solution, Figure S1) was chosen to obtain a satisfying signal-to-noise ratio under a lower reading voltage (−2.5 V). As shown in Figure 4b, the c-AFM mapping of the as-cast film shows no obvious signal above the noise background, corresponding to the iHRS measured in the devices (Figure S9). In contrast, there is a significant increase in the local current signal (Figure 4c) after thermal annealing, indicating the presence of CFs in the iLRS. When the PQDs were heated to over 50 °C, the weak acid–base interaction between PQDs and surface ligands could easily break, resulting in the detachment of ligands, leaving V_I_s in the original ligand sites^22,26,34,35^ to form CFs in iLRS. Additionally, the larger thermal energy at the elevated temperature makes it easier for the formation and migration of V_I_s. Lastly, the grazing-incidence wide-angle X-ray scattering patterns (GIWAXS, Figure S11) show a higher degree of crystallinity in the annealed film compared to the as-cast film, which may also aid the migration of V_I_s.^36,37^ In conclusion, the unusual initial LRS originates from the partial detachment of the surface ligand as a result of thermal annealing. On the other hand, no further growth of PQDs was observed due to the ligand detachment, as evidenced by the unchanged crystalline phase and optical bandgap from XRD, UV–vis, and PL results.

To directly visualize CF formation and growth during the two set processes, we performed consecutive c-AFM scans on the same sample area preset to HRS. A higher voltage (−8 V) than that used in the RS device set process was applied to induce the formation of as many CFs as possible. As shown in Figures 4d–h and S12, we overlaid current mappings (green region) and topography mappings (blue and white region) taken simultaneously under the contact mode, allowing us to identify the preferential sites for CF formation and growth.^38,39^ The topography images revealed large grains formed by the agglomeration of multiple PQDs, consistent with tapping-mode AFM (Figure 1i) and HRTEM images (Figure S13). During the first three scans (Figures 4d,e and S12a), the number of CFs increases preferentially at GBs, where V_I_s initially concentrate. As a result, the overall current distribution shifts to higher values (Figure 4i). In the subsequent three scans (Figures 4f,g and S12b), CFs grew significantly along the grain boundary and partially extended into the GIs, resulting in an asymmetric tail in the current distribution extending toward the high current region, as shown in Figure 4i. This suggests the growth of existing CFs with improved local conductivity. After six scans, CF growth saturated, as indicated by both the mapping (Figures 4h and S12c,d) and statistical results (Figure 4i), representing the complete CF growth at all possible locations: both GBs and GIs.

**Scheme 1:**
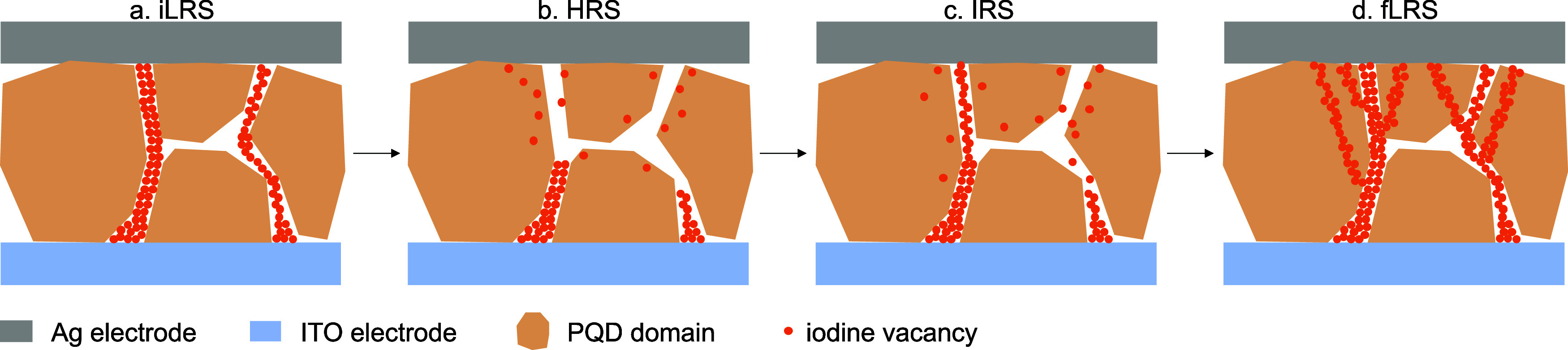
Schematic of CF Formation and Annihilation in the CsPbI_3_ PQD-Based Device in Different States; (a) iLRS; (b) HRS; (c) IRS; (d) fLRS

Based on these findings, we propose two pathways for the migration of V_I_s, namely, the grain boundary pathway and the grain interior pathway, to explain the ternary states observed in our RS devices, as summarized in Scheme 1. Initially, thermal annealing of the PQDs allows surface ligands to detach, which generates iodine vacancies that are preferentially located at GBs^40^ to form a few CFs, giving rise to iLRS (Scheme 1a). During the RESET process, the large current flowing through CF generates significant Joule heat, facilitating the lateral diffusion of iodine vacancies^41^ and CF annihilation, transitioning the device from iLRS to HRS (Scheme 1b). During the subsequent set process, more iodine vacancies are formed, which migrate vertically in response to the positive bias applied to the Ag electrode. Due to the lower formation and migration energy of V_I_s at GBs compared to GIs,^42,43^ those CFs primarily form and grow at GBs and then extend into the GIs at a larger bias, resulting in the consecutive transitions to IRS and fLRS (Scheme 1c,d). Compared to iLRS, more CFs are present in the fLRS state, as proven by the ex situ c-AFM mappings of the real device after a continuous *I*–*V* scan (compare Figures 4c and S14). This reduces the Joule heat generated within each individual CF, leading to the retention and further growth of CFs, thereby contributing to the observed WORM property.

At last, we discuss the impact of active layer materials and morphology on the RS behavior of our devices. In contrast to our optimized devices, RS devices based on the 3D CsPbI_3_ active layer show iHRS under negative bias and binary states under positive bias (Figure S15a). While the GIs of CsPbI_3_ PQDs are formed by weak agglomeration of individual PQDs,^44^ the GIs of 3D CsPbI_3_ are much more closely packed, hindering the growth of CFs. Similarly, devices based on CsPbBr_3_ PQDs also show binary states only (Figure S15b). We attributed this to the stronger electron-withdrawing ability of Br compared to I, resulting in a higher formation and migration energy of bromine vacancies.^45^ The grain size also plays an important role in the multilevel behavior of our RS devices, as demonstrated by CsPbI_3_ PQD-based devices with active layers deposited from either toluene or octane (Figure S16).

## 3 Conclusions

In summary, we have demonstrated the first application of CsPbI_3_ PQDs for WORM-type RS devices. The device exhibits remarkable intrinsic ternary states with an ON/OFF ratio of over 10^3^, long retention time of 10^4^ s, and good stability over 10 days, which is superior to most PQD-based devices. The unusual iLRS observed in our device was mainly attributed to the detachment of surface ligands during thermal annealing of the PQD layer, which increases the population of mobile iodine vacancies crucial for CF formation. The multilevel behavior under forward bias stemmed from the two distinct pathways (GBs and GIs) for iodine vacancy migration under electric bias, as directly visualized by in situ c-AFM. Our work underscores the potential of perovskite PQDs in fabricating low-cost and efficient RS devices with a simple device structure by rationally controlling the drift of mobile iodine vacancies. Lastly, the ion migration mechanism elucidated in this study may also be applicable to other vertical diode devices with similar structures, including perovskite photovoltaics and light-emitting diodes.

## 4 Experimental Section

### 4.1 Materials

Lead iodide (PbI_2_, 99.999%) was purchased from Xi’an Polymer Light Technology Crop. Lead bromide (PbBr_2_, 99.99%) was purchased from Greatcell Solar. ITO glass was purchased from Advanced Election Technology Co., Ltd. Cesium acetate (CsAc, 99.9%), oleic acid (OA, analytical reagent), oleylamine (OAm, technical grade 80–90%), 1-octadecene (ODE, technical grade 90%), and methyl acetate (MeOAc, anhydrous 99.5%) were purchased from Aladdin Bio-Chem Technology Crop. Toluene (anhydrous, 99.8%), hexane (99%), and anhydrous dimethylforamide (DMF) were purchased from Sigma-Aldrich. All of the chemicals were used without further purification.

### 4.2 Synthesis of PQDs

The CsPbI_3_ PQDs were synthesized by the hot-injection method according to the previously reported work.^23^ 5 mmol CsAc was dissolved in 10 mL OA and stirred at 90 °C. One mmol portion of PbI_2_ was dissolved in 10 mL of an ODE and stirred at 120 °C. After the Pb precursor reached 120 °C, 1.25 mL of OA and 1.25 mL of an OAm were added into the Pb precursor. Then, keep stirring in vacuum condition for 30 min. Ensuring all PbI_2_ was dissolved, the temperature was increased to 150 °C for 15 min. Using 0.4 mL of Cs precursor for hot injection and preparing ice water in advance. After injecting the Cs precursor into the Pb precursor, the PQD solution was obtained and quickly transported to ice water at the tenth second of the reaction.

Post-treatment was also needed for the purification of the PQDs. First, 30 mL of MeOAc was added to the PQD solution and centrifuged at 7500 rpm for 3 min. Then, precipitation was dissolved in 15 mL of hexane. After adding 18 mL of MeOAc to the solution, it was centrifuged at 7500 rpm for 3 min again. Using hexane to dissolve the precipitation again gave the PQD solution. After the washing process, the PQD solution was stored in the fridge overnight. Before use, the residuals and precipitation were removed by centrifugation at 4000 rpm for 3 min. PQDs were dried under vacuum conditions and redissolved in a mixed hexane and toluene (1:1 vol %) solvent.

The CsPbBr_3_ PQDs were synthesized using the same method as CsPbI_3_ PQDs, with PbBr_2_ taking the place of PbI_2_.

### 4.3 Device Fabrication

Cleaned patterned ITO substrates were treated with ultraviolet ozone for 30 min. Then, the PQD solution was spin-coated directly on the ITO substrates at 500 rpm for 3 s, 1500 rpm for 15 s, and 2000 rpm for 10 s, followed by 100 °C thermal annealing for 10 min. At last, a 60 nm Ag electrode layer was deposited by thermal evaporation.

For 3D CsPbI_3_, 0.6 mmol CsI, 0.6 mmol PbI_2_, and 0.3 mmol DMAI were dissolved in 1 mL DMF. The perovskite precursor was spin-coated directly on the preheated ITO substrate at 3000 rpm for 30 s, followed by 2 min thermal annealing at 150 °C.

### 4.4 Characterizations

PQD TEM images and size distribution were recorded with an FEI TS12. PerkinElmer Lambda 950 was used to measure the UV–vis absorption spectra of the PQDs in solution and thin film states. The crystalline structure was characterized by XRD (Rigaku X-ray diffractometer). The cross-sectional sample of RS devices was fabricated using the Thermo Scientific FIB, which was then characterized by HRTEM (FEI TF20) and STEM-EDXS to determine PQD alignments and elemental distribution. The surface morphology was measured using an SEM (FEI QF400) with a 5.0 keV acceleration voltage. The cross-sectional image was taken by a Thermo Scientific FIB. The IV curves and other electric characterizations were measured by a Keysight B2901A source meter unit. The temperature-dependent resistance was performed by a Keithley 2612 dual-channel source meter. A Scientific Instruments 9700 temperature controller was used to control the test temperature from 243.15 to 293.15 K. GIWAXS was carried out using Xeuss 2.0 SAXS/WAXS laboratory beamline with a Cu X-ray source (8.05 keV, 1.54 Å) and a Pilatus3R 300 K detector. The incident angle is 0.1°. AFM was conducted using a JPK NanoWizard NanoOptics from Bruker. Topography images were taken under the tapping mode, and c-AFM images were taken under the contact mode via the conductive ElectriCont-G probe with Pt overall coating from Budget Sensors. XPS measurements were performed at the BL09A2 U5 beamline at the National Synchrotron Radiation Research Centre, Taiwan. The incident photon energy was 750 eV, and the data was calibrated by the position of the C 1s peak at 284.8 eV. The Renishaw inVia Qontor Micro Raman was used to measure PL spectroscopy with a 532 nm excitation laser.

## Supporting Information Available

*I*–*V* curves; device statistic data; AFM, c-AFM, and HRTEM images; GIWAXS patterns; and PL (PDF)

## References

[ref1] Chen A. A Review of Emerging Non-Volatile Memory (NVM) Technologies and Applications. Solid-State Electron. 2016, 125, 25–38. 10.1016/j.sse.2016.07.006.

[ref2] Möller S.; Perlov C.; Jackson W.; Taussig C.; Forrest S. R. A Polymer/Semiconductor Write-Once Read-Many-Times Memory. Nat. 2003, 426 (6963), 166–169. 10.1038/nature02070. 14614502

[ref3] Hsu C.-C.; Cheng C.-W.; Jhang W.-C.; Wen S.-M. Modification of OFF-and ON-Resistances of SrTiOx WORM Memories by Thermal Annealing Processes. IEEE Trans. Electron Devices 2022, 69 (3), 1020–1027. 10.1109/TED.2021.3139855.

[ref4] Hsu C.-C.; Tsai J.-E.; Lin Y.-S. A write-once-read-many-times memory based on a sol-gel derived copper oxide semiconductor. Physica B: Condensed Matter 2019, 562, 20–25. 10.1016/j.physb.2019.03.007.

[ref5] Wang Y.; Lv Z.; Liao Q.; Shan H.; Chen J.; Zhou Y.; Zhou L.; Chen X.; Roy V. A.; Wang Z.; Xu Z.; Zeng Y.-J.; Han S.-T. Synergies of Electrochemical Metallization and Valance Change in All-Inorganic Perovskite Quantum Dots for Resistive Switching. Adv. Mater. 2018, 30 (28), 1800327 10.1002/adma.201800327. 29782667

[ref6] Das U.; Sarkar P. K.; Das D.; Paul B.; Roy A. Influence of Nanoscale Charge Trapping Layer on the Memory and Synaptic Characteristics of a Novel Rubidium Lead Chloride Quantum Dot Based Memristor. Adv. Electron. Mater. 2022, 8 (5), 2101015 10.1002/aelm.202101015.

[ref7] Upadhyay N. K.; Sun W.; Lin P.; Joshi S.; Midya R.; Zhang X.; Wang Z.; Jiang H.; Yoon J. H.; Rao M.; Chi M.; Xia Q.; Yang J. J. A Memristor with Low Switching Current and Voltage for 1S1R Integration and Array Operation. Adv. Electron. Mater. 2020, 6 (5), 1901411 10.1002/aelm.201901411.

[ref8] Park S.-O.; Jeong H.; Park J.; Bae J.; Choi S. Experimental Demonstration of Highly Reliable Dynamic Memristor for Artificial Neuron and Neuromorphic Computing. Nat. Commun. 2022, 13 (1), 2888 10.1038/s41467-022-30539-6. 35660724 PMC9166790

[ref9] Sakhatskyi K.; John R. A.; Guerrero A.; Tsarev S.; Sabisch S.; Das T.; Matt G. J.; Yakunin S.; Cherniukh I.; Kotyrba M.; Berezovska Y.; Bodnarchuk M. I.; Chakraborty S.; Bisquert J.; Kovalenko M. V. Assessing the Drawbacks and Benefits of Ion Migration in Lead Halide Perovskites. ACS Energy Lett. 2022, 7 (10), 3401–3414. 10.1021/acsenergylett.2c01663. 36277137 PMC9578653

[ref10] Tress W. Metal Halide Perovskites as Mixed Electronic–Ionic Conductors: Challenges and Opportunities From Hysteresis to Memristivity. J. Phys. Chem. Lett. 2017, 8 (13), 3106–3114. 10.1021/acs.jpclett.7b00975. 28641009

[ref11] Zhu X.; Lu W. D. Optogenetics-Inspired Tunable Synaptic Functions in Memristors. ACS Nano 2018, 12 (2), 1242–1249. 10.1021/acsnano.7b07317. 29357245

[ref12] Wu T.; Mukherjee R.; Ovchinnikova O. S.; Collins L.; Ahmadi M.; Lu W.; Kang N.-G.; Mays J. W.; Jesse S.; Mandrus D.; Hu B. Metal/Ion Interactions Induced p–i–n Junction in Methylammonium Lead Triiodide Perovskite Single Crystals. J. Am. Chem. Soc. 2017, 139 (48), 17285–17288. 10.1021/jacs.7b10416. 29137455

[ref13] Walsh A.; Stranks S. D. Taking Control of Ion Transport in Halide Perovskite Solar Cells. ACS Energy Lett. 2018, 3 (8), 1983–1990. 10.1021/acsenergylett.8b00764.

[ref14] Yoo E. J.; Lyu M.; Yun J. H.; Kang C. J.; Choi Y. J.; Wang L. Resistive Switching Behavior in Organic–Inorganic Hybrid CH3NH3PbI3–xClx Perovskite for Resistive Random Access Memory Devices. Adv. Mater. 2015, 27 (40), 6170–6175. 10.1002/adma.201502889. 26331363

[ref15] De Roo J.; Ibáñez M.; Geiregat P.; Nedelcu G.; Walravens W.; Maes J.; Martins J. C.; Van Driessche I.; Kovalenko M. V.; Hens Z. Highly Dynamic Ligand Binding and Light Absorption Coefficient of Cesium Lead Bromide Perovskite Nanocrystals. ACS Nano 2016, 10 (2), 2071–2081. 10.1021/acsnano.5b06295. 26786064

[ref16] Ye J.; Byranvand M. M.; Martínez C. O.; Hoye R. L.; Saliba M.; Polavarapu L. Defect Passivation in Lead-Halide Perovskite Nanocrystals and Thin Films: toward Efficient LEDs and Solar Cells. Angew. Chem. 2021, 133 (40), 21804–21828. 10.1002/ange.202102360. PMC8518834 33730428

[ref17] Swarnkar A.; Marshall A. R.; Sanehira E. M.; Chernomordik B. D.; Moore D. T.; Christians J. A.; Chakrabarti T.; Luther J. M. Quantum Dot–Induced Phase Stabilization of α-CsPbI3 Perovskite for High-Efficiency Photovoltaics. Science 2016, 354 (6308), 92–95. 10.1126/science.aag2700. 27846497

[ref18] Liu X.; Ren S.; Li Z.; Guo J.; Yi S.; Yang Z.; Hao W.; Li R.; Zhao J. Flexible Transparent High-Efficiency Photoelectric Perovskite Resistive Switching Memory. Adv. Funct. Mater. 2022, 32, 2202951 10.1002/adfm.202202951.

[ref19] Jiang Q.; Ren Y.; Cui Z.; Li Z.; Hu L.; Guo R.; Duan S.; Xie F.; Zhou G.; Xiong S. CsPbBr3 Perovskite Quantum Dots Embedded in Polystyrene-poly2-vinyl Pyridine Copolymer for Robust and Light-Tunable Memristors. ACS Appl. Nano Mater. 2023, 6 (10), 8655–8667. 10.1021/acsanm.3c00975.

[ref20] An H.; Kim W. K.; Wu C.; Kim T. W. Highly-Stable Memristive Devices Based on Poly (methylmethacrylate): CsPbCl3 Perovskite Quantum Dot Hybrid Nanocomposites. Org. Electron. 2018, 56, 41–45. 10.1016/j.orgel.2018.02.001.

[ref21] Yang K.; Li F.; Veeramalai C. P.; Guo T. A Facile Synthesis of CH3NH3PbBr3 Perovskite Quantum Dots and Their Application in Flexible Nonvolatile Memory. Appl. Phys. Lett. 2017, 110 (8), 083102 10.1063/1.4976709.

[ref22] Muthu C.; Resmi A.; Pious J. K.; Dayal G.; Krishna N.; Jinesh K.; Vijayakumar C. Resistive Switching in Formamidinium Lead Iodide Perovskite Nanocrystals: a Contradiction to the Bulk Form. J. Mater. Chem. C 2021, 9 (1), 288–293. 10.1039/D0TC03275A.

[ref23] Chen K.; Jin W.; Zhang Y.; Yang T.; Reiss P.; Zhong Q.; Bach U.; Li Q.; Wang Y.; Zhang H.; Bao Q.; Liu Y. High Efficiency Mesoscopic Solar Cells Using CsPbI3 Perovskite Quantum Dots Enabled by Chemical Interface Engineering. J. Am. Chem. Soc. 2020, 142 (8), 3775–3783. 10.1021/jacs.9b10700. 31967471

[ref24] Sanehira E. M.; Marshall A. R.; Christians J. A.; Harvey S. P.; Ciesielski P. N.; Wheeler L. M.; Schulz P.; Lin L. Y.; Beard M. C.; Luther J. M. Enhanced mobility CsPbI3 quantum dot arrays for record-efficiency, high-voltage photovoltaic cells. Sci. Adv. 2017, 3 (10), eaao4204 10.1126/sciadv.aao4204. 29098184 PMC5659658

[ref25] Tavakoli M. M.; Nasilowski M.; Zhao J.; Bawendi M. G.; Kong J. Efficient Semitransparent CsPbI3 Quantum Dots Photovoltaics Using a Graphene Electrode. Small Methods 2019, 3 (12), 1900449 10.1002/smtd.201900449.

[ref26] Li Y.; Qin M.; Wang Y.; Li S.; Qin Z.; Tsang S.-W.; Su C.-J.; Ke Y.; Lu X. Controllable Black-to-Yellow Phase Transition by Tuning the Lattice Symmetry in Perovskite Quantum Dots. Small 2023, 19 (47), 2303885 10.1002/smll.202303885. 37496030

[ref27] Zhao Q.; Hazarika A.; Schelhas L. T.; Liu J.; Gaulding E. A.; Li G.; Zhang M.; Toney M. F.; Sercel P. C.; Luther J. M. Size-Dependent Lattice Structure and Confinement Properties in CsPbI3 Perovskite Nanocrystals: Negative Surface Energy for Stabilization. ACS Energy Lett. 2020, 5 (1), 238–247. 10.1021/acsenergylett.9b02395.

[ref28] Gonzales C.; Guerrero A.; Bisquert J. Spectral Properties of the Dynamic State Transition in Metal Halide Perovskite-Based Memristor Exhibiting Negative Capacitance. Appl. Phys. Lett. 2021, 118 (7), 073501 10.1063/5.0037916.

[ref29] Jiang X.; Zhao Y.; Chen Y.; Li D.; Luo Y.; Zhao D.; Sun Z.; Sun J.; Zhao H. Characteristics of Different Types of Filaments in Resistive Switching Memories Investigated by Complex Impedance Spectroscopy. Appl. Phys. Lett. 2013, 102 (25), 253507 10.1063/1.4812811.

[ref30] Berruet M.; Pérez-Martínez J. C.; Romero B.; Gonzales C.; Al-Mayouf A. M.; Guerrero A.; Bisquert J. Physical Model for the Current–Voltage Hysteresis and Impedance of Halide Perovskite Memristors. ACS Energy Lett. 2022, 7 (3), 1214–1222. 10.1021/acsenergylett.2c00121.

[ref31] Haruyama J.; Sodeyama K.; Han L.; Tateyama Y. First-Principles Study of Ion Diffusion in Perovskite Solar Cell Sensitizers. J. Am. Chem. Soc. 2015, 137 (32), 10048–10051. 10.1021/jacs.5b03615. 26258577

[ref32] Eames C.; Frost J. M.; Barnes P. R.; O’regan B. C.; Walsh A.; Islam M. S. Ionic Transport in Hybrid Lead Iodide Perovskite Solar Cells. Nat. Commun. 2015, 6 (1), 7497 10.1038/ncomms8497. 26105623 PMC4491179

[ref33] Miao F.; Strachan J. P.; Yang J. J.; Zhang M. X.; Goldfarb I.; Torrezan A. C.; Eschbach P.; Kelley R. D.; Medeiros-Ribeiro G.; Williams R. S. Anatomy of a Nanoscale Conduction Channel Reveals the Mechanism of a High-Performance Memristor. Adv. Mater. 2011, 23 (47), 5633–5640. 10.1002/adma.201103379. 22065427

[ref34] Lim S.; Han S.; Kim D.; Min J.; Choi J.; Park T. Key Factors Affecting the Stability of CsPbI3 Perovskite Quantum Dot Solar Cells: a Comprehensive Review. Adv. Mater. 2022, 35, 2203430 10.1002/adma.202203430. 35700966

[ref35] Boote B. W.; Andaraarachchi H. P.; Rosales B. A.; Blome-Fernández R.; Zhu F.; Reichert M. D.; Santra K.; Li J.; Petrich J. W.; Vela J.; Smith E. A. Unveiling the Photo-and Thermal-Stability of Cesium Lead Halide Perovskite Nanocrystals. ChemPhysChem 2019, 20 (20), 2647–2656. 10.1002/cphc.201900432. 31441207

[ref36] Li Y.; Zhang C.; Shi Z.; Ma C.; Wang J.; Zhang Q. Recent Advances on Crystalline Materials-based Flexible Memristors for Data Storage and Neuromorphic Applications. Sci. China: Mater. 2021, 65, 2110–2127. 10.1007/s40843-021-1771-5.

[ref37] Kim S. J.; Lee T. H.; Yang J.-M.; Yang J. W.; Lee Y. J.; Choi M.-J.; Lee S. A.; Suh J. M.; Kwak K. J.; Baek J. H.; Im I. H.; Lee D. E.; Kim J. Y.; Kim J.; Han J. S.; Kim S. Y.; Lee D.; Park N.-G.; Jang H. W. Vertically Aligned Two-dimensional Halide Perovskites for Reliably Operable Artificial Synapses. Mater. Today 2022, 52, 19–30. 10.1016/j.mattod.2021.10.035.

[ref38] Lanza M.; Bersuker G.; Porti M.; Miranda E.; Nafría M.; Aymerich X. Resistive Switching in Hafnium Dioxide Layers: Local Phenomenon at Grain Boundaries. Appl. Phys. Lett. 2012, 101 (19), 193502 10.1063/1.4765342.

[ref39] Lanza M.; Zhang K.; Porti M.; Nafría M.; Shen Z.; Liu L.; Kang J.; Gilmer D.; Bersuker G. Grain Boundaries as Preferential Sites for Resistive Switching in the HfO2 Resistive Random Access Memory Structures. Appl. Phys. Lett. 2012, 100 (12), 123508 10.1063/1.3697648.

[ref40] Alosaimi G.; Huang C.-Y.; Sharma P.; Wu T.; Seidel J. Morphology-Dependent Charge Carrier Dynamics and Ion Migration Behavior of CsPbBr3 Halide Perovskite Quantum Dot Films. Small 2023, 19 (20), 2207220 10.1002/smll.202207220. 36807547

[ref41] Strukov D. B.; Alibart F.; Stanley Williams R. Thermophoresis/Diffusion as A Plausible Mechanism for Unipolar Resistive Switching in Metal–Oxide–Metal Memristors. Appl. Phys. A: Mater. Sci. Process. 2012, 107 (3), 509–518. 10.1007/s00339-012-6902-x.

[ref42] McGovern L.; Koschany I.; Grimaldi G.; Muscarella L. A.; Ehrler B. Grain Size Influences Activation Energy and Migration Pathways in MAPbBr3 Perovskite Solar Cells. J. Phys. Chem. Lett. 2021, 12 (9), 2423–2428. 10.1021/acs.jpclett.1c00205. 33661008 PMC8041307

[ref43] Shao Y.; Fang Y.; Li T.; Wang Q.; Dong Q.; Deng Y.; Yuan Y.; Wei H.; Wang M.; Gruverman A.; Shield J.; Huang J. Grain boundary dominated ion migration in polycrystalline organic–inorganic halide perovskite films. Energy Environ. Sci. 2016, 9 (5), 1752–1759. 10.1039/C6EE00413J.

[ref44] Li S.; Wang Z.; Li Y.; Su C.-J.; Fu Y.; Wang Y.; Lu X. Fostering the Dense Packing of Halide Perovskite Quantum Dots through Binary-Disperse Mixing. ACS Nano 2023, 17 (20), 20634–20642. 10.1021/acsnano.3c07688. 37787473 PMC10604077

[ref45] Nenon D. P.; Pressler K.; Kang J.; Koscher B. A.; Olshansky J. H.; Osowiecki W. T.; Koc M. A.; Wang L.-W.; Alivisatos A. P. Design Principles for Trap-Free CsPbX3 Nanocrystals: Enumerating and Eliminating Surface Halide Vacancies with Softer Lewis Bases. J. Am. Chem. Soc. 2018, 140 (50), 17760–17772. 10.1021/jacs.8b11035. 30501174

