# Supplementary material for: CsPbI3 Perovskite Quantum Dot-Based WORM Memory Device with Intrinsic Ternary States
Source: ACS Appl Mater Interfaces. 2024 Jul 22;16(30):39827–34. doi: 10.1021/acsami.4c07044 (PMC11299139; doi:10.1021/acsami.4c07044)
Supplement: Supplementary file 1 — am4c07044_si_001.pdf [file am4c07044_si_001.pdf]

Supporting Information

$CsPbI_3$ perovskite quantum dot-based WORM memory device with intrinsic ternary states

Luhang Xu[1], Yuang Fu[1], Yuhao Li[2], Guodong Zhou[3], and Xinhui Lu[1,]*

[1]Department of Physics, The Chinese University of Hong Kong, New Territories 999077, Hong Kong SAR, China.

[2]Spallation Neutron Source Science Center, Dongguan 523803, China.

[3]College of Integrated Circuits, Zhejiang University, Hangzhou 311200, China

Corresponding Author

*Xinhui Lu – Email: xinhui.lu@cuhk.edu.hk

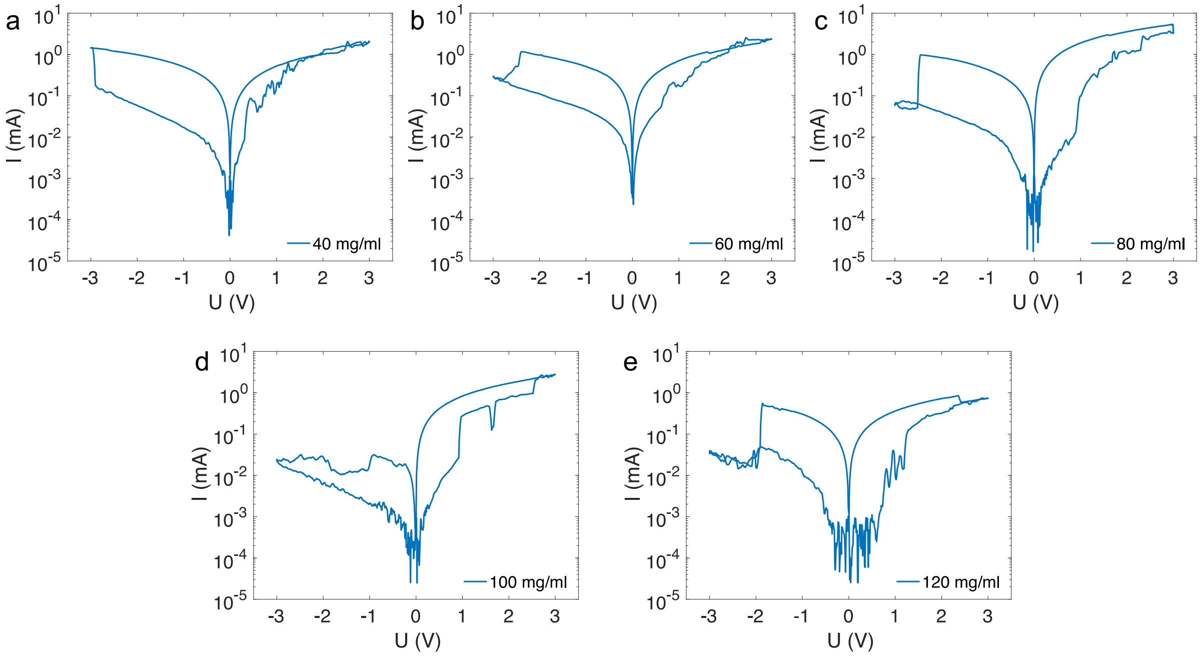

Figure S1. I-V characteristics of Ag/$CsPbI_3$ PQD/ITO device with different thicknesses of PQD layer controlled by the concentration of PQD solution: (a) 40, (b) 60, (c) 80 (optimal), (d) 100, and (e) 120 mg/ml.

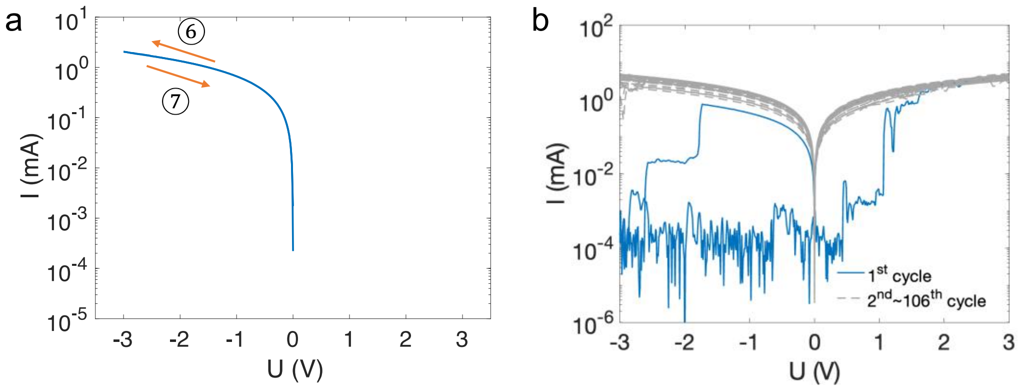

Figure S2. (a) I-V characteristics of Ag/$CsPbI_3$ PQDs/ITO device after the first voltage loop shown in **Figure 2c**. The voltage applied to the Ag electrode was swept from 0→-3V→0, corresponding to the RESET process in the first loop. (b) The 100 cycles of the I-V curve of the $CsPbI_3$ PQD-based WORM device.

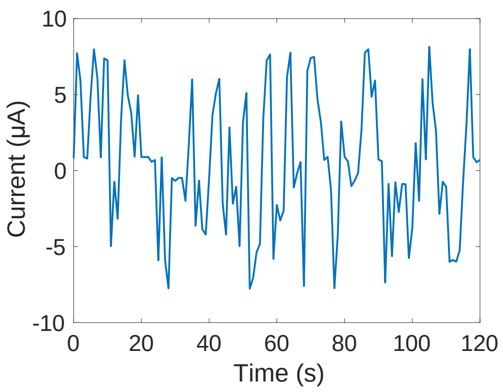

Figure S3. The noise level of the source meter under the open circuit condition.

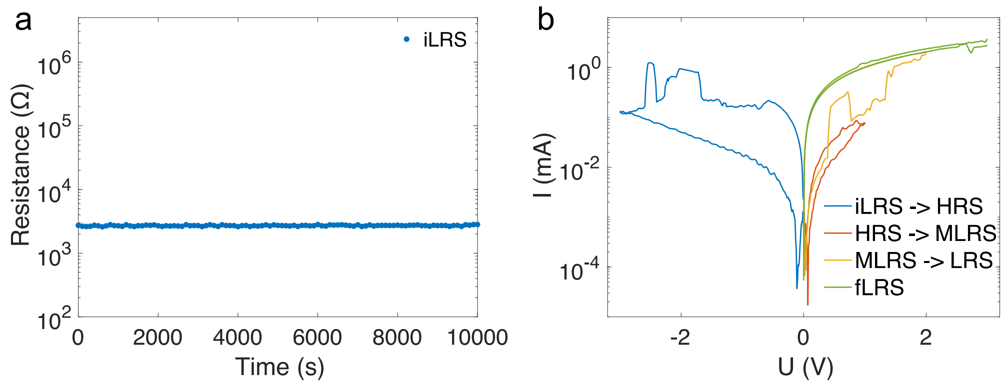

Figure S4. (a) The retention time of iLRS read by a small reading voltage (0.1 V). (b) The continuous I-V curve of the device for retention time characterization.

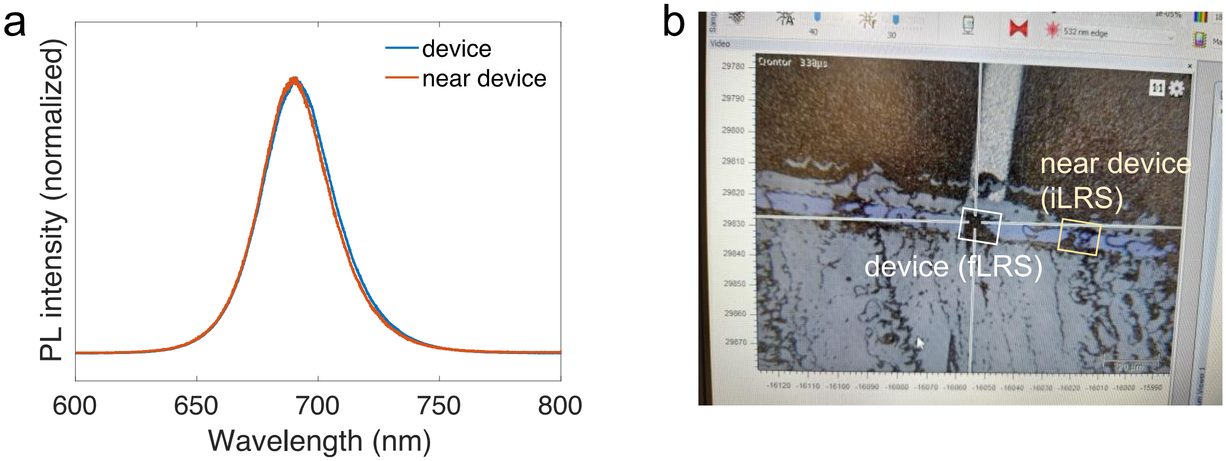

Figure S5. (a) Micro-PL spectroscopy of the PQD active layer in the RS device at fLRS state after peeling off the Ag electrode. As shown in (b) the live optical image, the measurements were taken in the area close to the device (yellow box) where the PQD layer remained in the iLRS state and in the device area (white box) where the PQD layer transited to the fLRS after the Set/Reset cycle. The invariant peak positions and widths suggest that the PQDs do not grow or undergo structural change during state transitions.

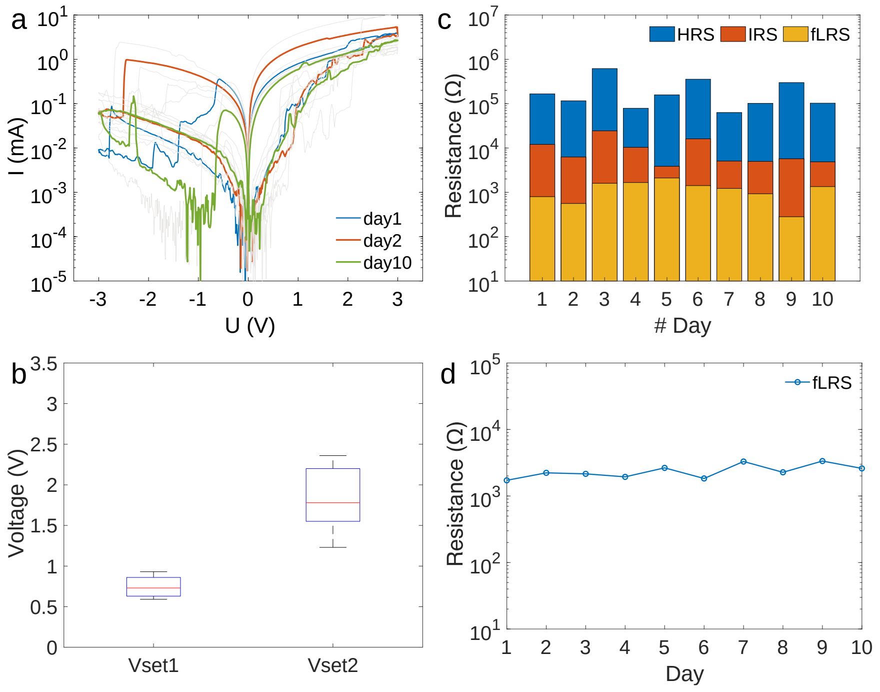

Figure S6. Stability of the RS devices during its 10 days storage in a glovebox filled with N_2. (a) Characteristic I-V curves of the device on days 1, 2, and 10. (b) Statistic data of voltage distributions for the two set processes. (c) Resistances of HRS, IRS, and LRS on different days. (d) fLRS stability of one device after transition for 10 days.

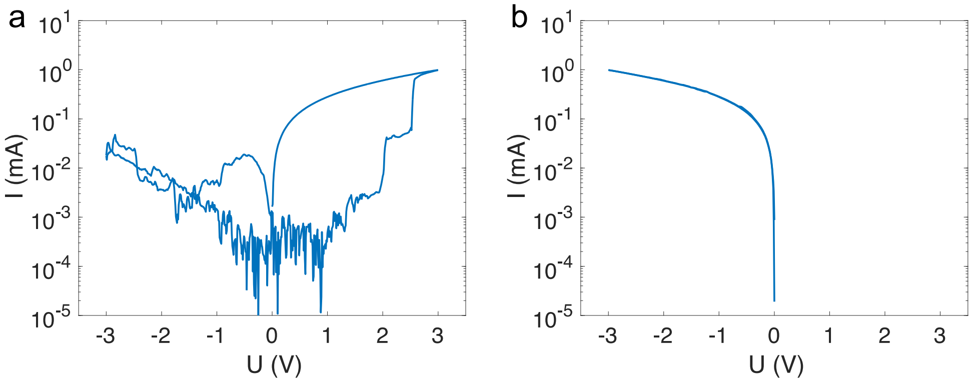

Figure S7. I-V characteristics of the Au/$CsPbI_3$ PQDs/ITO device: (a) the first cycle following the 0 V→-3 V→0 V→+3 V→0 V voltage loop; (b) the second cycle following the 0 V→-3 V→0 V voltage loop. In all I-V measurements, the ITO substrate was grounded, and the bias was applied to the Au top electrode.

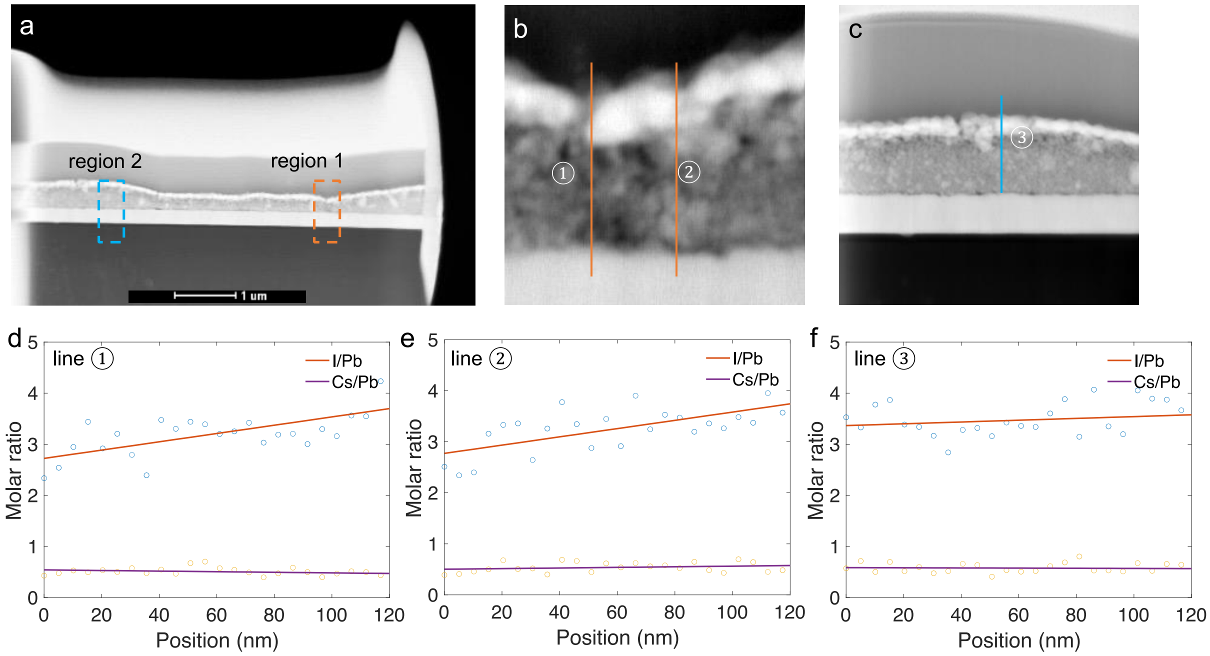

Figure S8. (a) The scanning transmission electron microscopy (STEM) image of the device cross-section. The device was set to the fLRS, and the sample was fabricated by the FIB technique. The two regions where EDXS line scans were taken were highlighted as Regions 1 and 2, respectively with their enlarged STEM images shown in (b) and (c). The EDXS line scans in different regions: (d) line 1 in Region 1, (e) line 2 in Region 1, and (f) line 3 in Region 2. The Cs/Pb ratio is less than unity due to the insufficient signal intensity caused by the main peak of the Cs partially overlapping with the minor peak of Iodine.

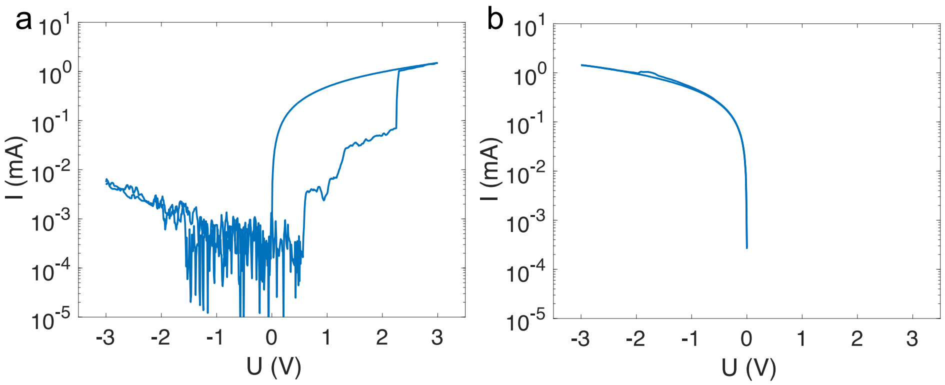

Figure S9. I-V characteristics of Ag/as-cast $CsPbI_3$ PQDs/ITO device: (a) the first cycle following the 0→-3V→0→+3V→0 voltage loop; (b) the second cycle following the 0→-3V→0 voltage loop.

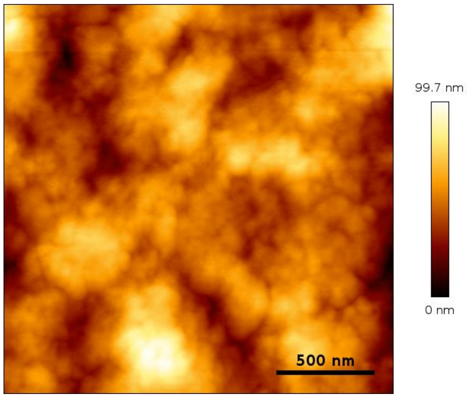

Figure S10. Topography images of as-cast $CsPbI_3$ PQD film surface measured by tapping mode AFM.

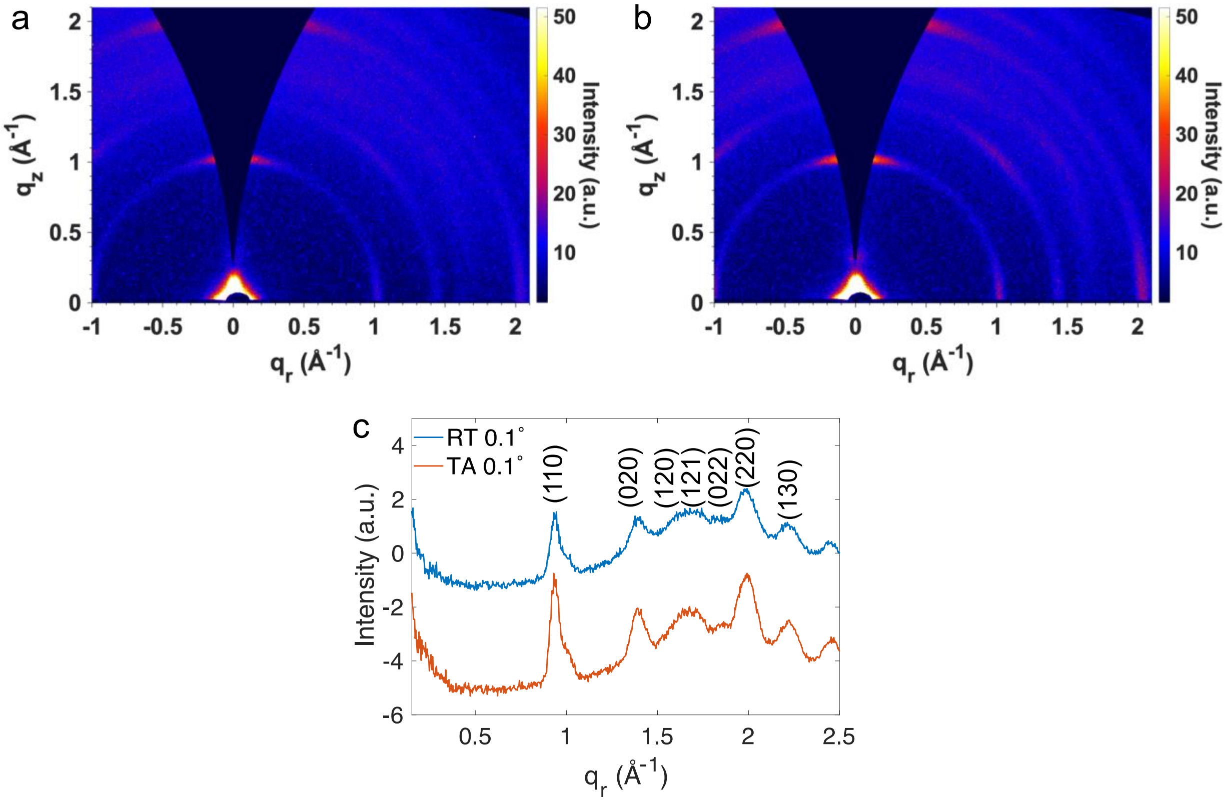

Figure S11. GIWAXS pattern: the $CsPbI_3$ PQD film (a) without and (b) with thermal annealing at 100 °C for 10 mins. The corresponding sector intensity profiles are shown in (c). The incident angle used was 0.1°.

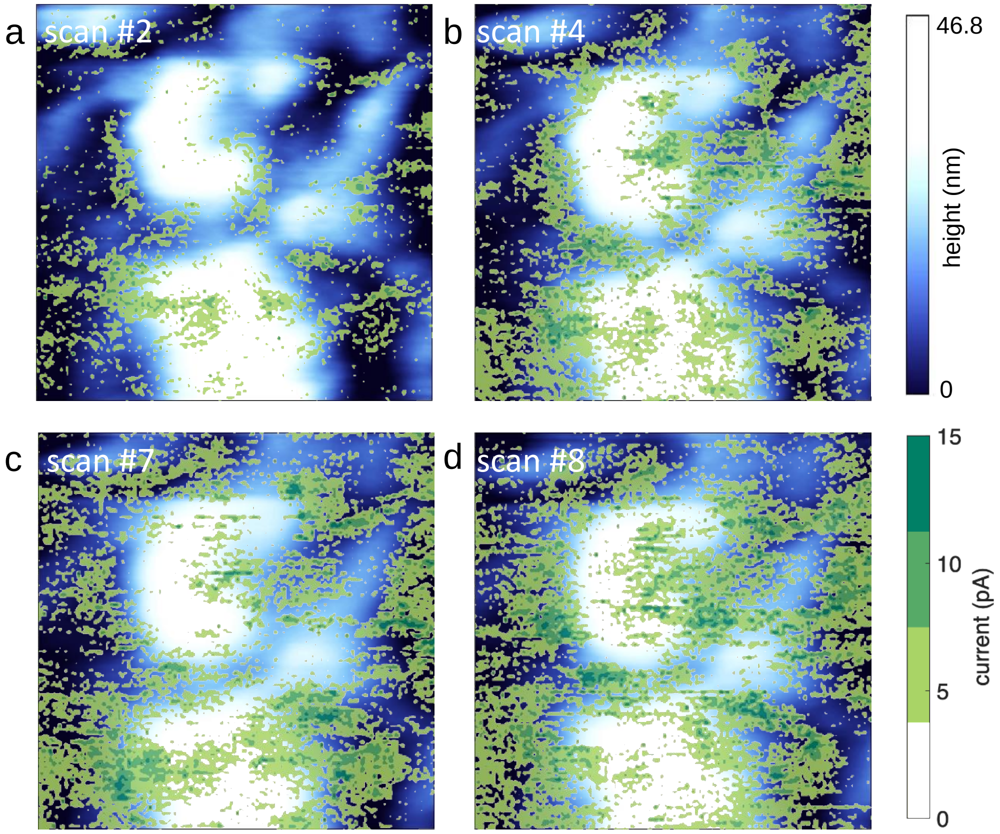

Figure S12. The AFM topography images (blue) overlapped with current mappings (green) taken during the (a) second, (b) fourth, (c) seventh, and (d) eighth scan.

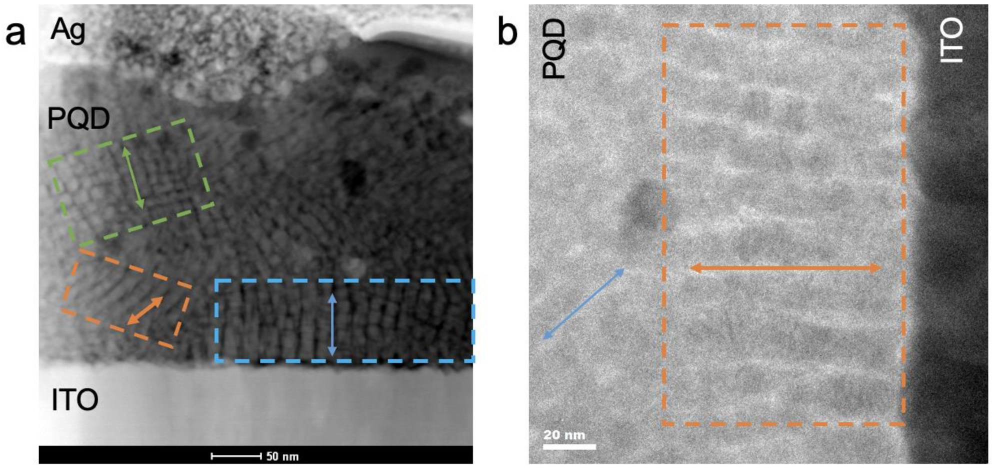

Figure S13. (a) The STEM image shows the cross-section of the RS device. (b) The HRTEM image of the same sample. Note that the reverse contrast observed in the two images was due to the different locations of the detectors in those two modes.

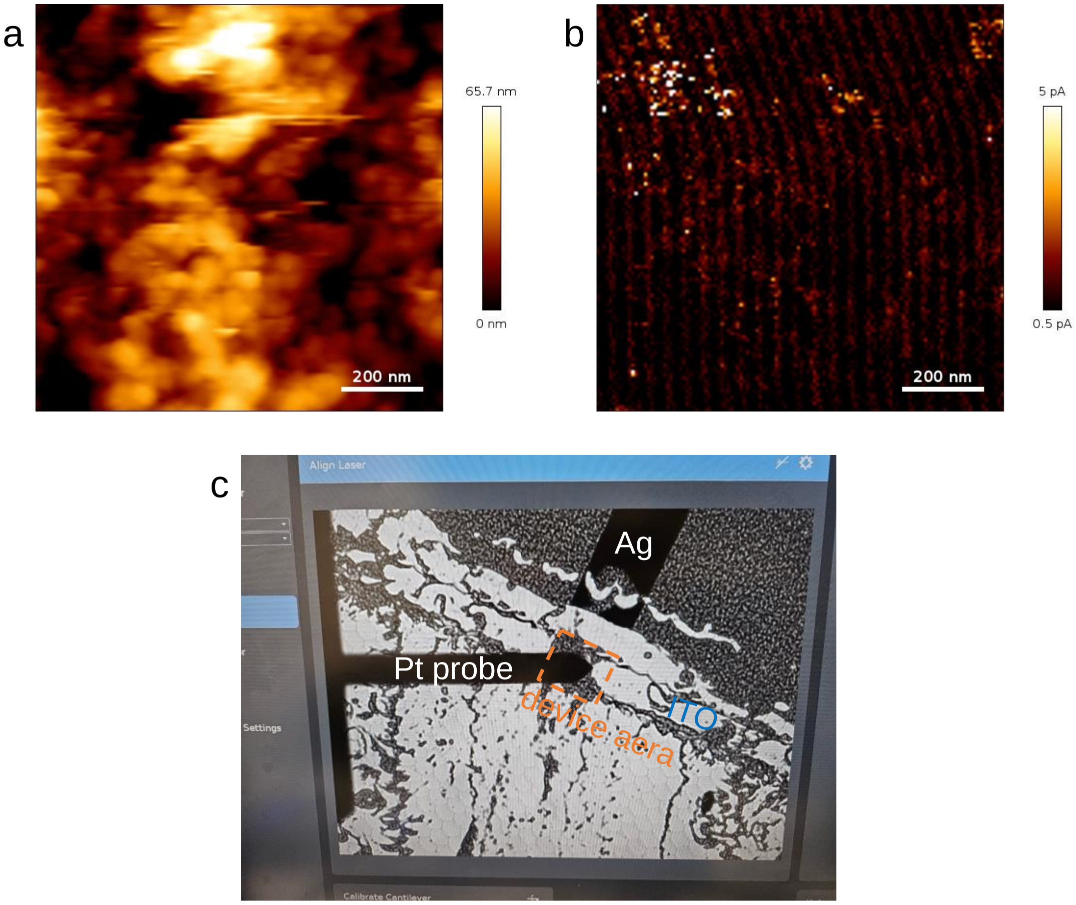

Figure S14. (a) The topography and (b) the current mapping (1μm×1μm) of the $CsPbI_3$ QD film in the device area. (c) The live optical image of the scanned area. The orange dash box points out the device area. The device was first set to fLRS by continuous I-V scanning, then the Ag electrode was peeled off for the clear filament observation. Compared to **Figure 4c** (2μm×2μm), the increase in filament population is clear.

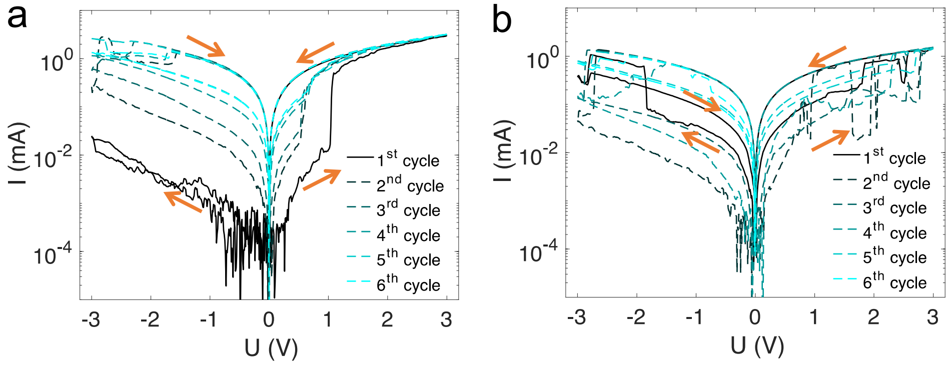

Figure S15. I-V characteristics of (a) the Ag/3D $CsPbI_3$/ITO device and (b) the Ag/$CsPbBr_3$ QDs/ITO device.

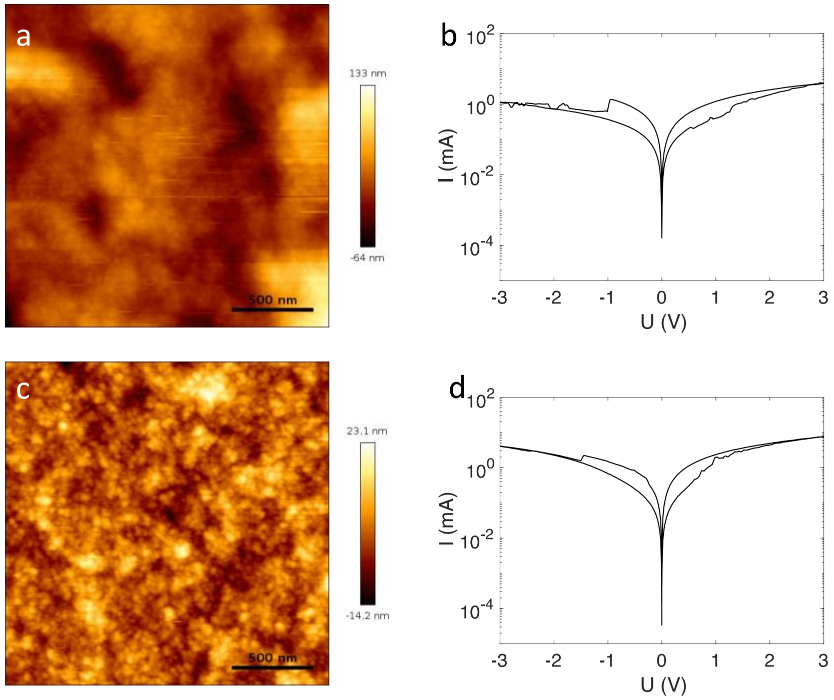

Figure S16. The topography images scanned by AFM of (a) the PQD film using pure toluene and (c) the PQD film using pure octane. (b) and (d) are device performance corresponding to (a) and (c), respectively.
